# Capturing Expert Knowledge for the Personalization of Cognitive Rehabilitation: Study Combining Computational Modeling and a Participatory Design Strategy

**DOI:** 10.2196/10714

**Published:** 2018-12-06

**Authors:** Ana Lúcia Faria, Maria Salomé Pinho, Sergi Bermúdez i Badia

**Affiliations:** 1 Madeira Interactive Technologies Institute Funchal Portugal; 2 Faculdade de Psicologia e de Ciências da Educação Universidade de Coimbra Coimbra Portugal; 3 Laboratório de Memória, Linguagem e Funções Executivas Coimbra Portugal; 4 Centro de Ciências Exatas e da Engenharia Universidade da Madeira Funchal Portugal

**Keywords:** stroke rehabilitation, attention, memory, executive function, language, cognition, community-based participatory research, patient-specific modeling

## Abstract

**Background:**

Cognitive impairments after stroke are not always given sufficient attention despite the critical limitations they impose on activities of daily living (ADLs). Although there is substantial evidence on cognitive rehabilitation benefits, its implementation is limited because of time and human resource’s demands. Moreover, many cognitive rehabilitation interventions lack a robust theoretical framework in the selection of paper-and-pencil tasks by the clinicians. In this endeavor, it would be useful to have a tool that could generate standardized paper-and-pencil tasks, parameterized according to patients' needs.

**Objective:**

In this study, we aimed to present a framework for the creation of personalized cognitive rehabilitation tasks based on a participatory design strategy.

**Methods:**

We selected 11 paper-and-pencil tasks from standard clinical practice and parameterized them with multiple configurations. A total of 67 tasks were assessed according to their cognitive demands (attention, memory, language, and executive functions) and overall difficulty by 20 rehabilitation professionals.

**Results:**

After assessing the internal consistency of the data—that is, *alpha* values from .918 to .997—we identified the parameters that significantly affected cognitive functions and proposed specific models for each task. Through computational modeling, we operationalized the tasks into their intrinsic parameters and developed a Web tool that generates personalized paper-and-pencil tasks—the Task Generator (TG).

**Conclusions:**

Our framework proposes an objective and quantitative personalization strategy tailored to each patient in multiple cognitive domains (attention, memory, language, and executive functions) derived from expert knowledge and materialized in the TG app, a cognitive rehabilitation Web tool.

## Introduction

### Background

Stroke is one of the most common causes of adult disability, and because of the aging of the population, the number of people having a stroke continues to rise. According to the 2015 Global Burden of Disease study, the total number of stroke events in Europe is predicted to increase by 34% between 2015 and 2035. This increasing number of people living with the effects of stroke results in a growing burden on families, societies, and health care systems. Reducing the long-term disability will help to bring down these costs [1].

### Cognitive and Motor Impairments After Stroke

Poststroke impairments impact the individual’s ability to safely and independently carry out activities of daily living (ADLs) and to restart prestroke personal, social, and vocational activities. Stroke survivors often express that they feel like a different person, not because of the typical motor sequels but because of changes they suffer in cognitive functions underlying their capacity for language, attention, executive functions, and memory [2].

Currently, rehabilitation following stroke routinely takes a bottom-up approach, with the primary focus placed on motor gait retraining, followed by upper limb rehabilitation and speech and language therapy [3]. Consequently, cognitive impairments are not always systematically assessed and treated. Moreover, current rehabilitation entails a high demand for human resources, making them time consuming and expensive. As a result, there is a high number of patients per therapist that makes it challenging to deliver a rehabilitation program with the appropriate intensity and training, hampering the recovery potential for some patients [4]. It is known that inappropriate cognitive rehabilitation limits patients’ capacity of living independently. In fact, it has been shown that the level of cognitive impairment correlates with the length of inpatient stay and the number and frequency of referrals for outpatient and home therapies [5].

In a recent James Lind Alliance study, 799 stroke survivors were interviewed about their unmet needs following a stroke, and they reported problems with concentration (45%), memory (43%), and reading (23%) [6]. A high proportion felt that issues such as memory and concentration had not been addressed appropriately, especially when compared with other issues such as mobility. Similarly, when caregivers and health professionals were consulted, the main conclusion of the study was that investigating ways to improve cognition after stroke should be a research priority [7]. There is, therefore, an avoidable psychosocial and economic cost derived from the currently limited cognitive rehabilitation, which contributes to the patient's increased dependency on relatives, professionals, and health care systems and their premature placement at nursing homes [8].

### Cognitive Rehabilitation and What Are We Missing?

Rehabilitation refers to the act of relearning a previously learned behavior that has been disrupted by brain damage. It involves re-establishing connection weights or synapses within the network, diverting the information by building new connection weights or synapses or activating the neurons that were not previously used [9]. Ben-Yishay and Prigatano defined cognitive rehabilitation as “the amelioration of deficits in problem-solving abilities to improve functional competence in everyday situations” [10]. The main point about this definition is the understanding that cognitive rehabilitation should focus on real-life functional problems. In rehabilitation, models and theories are useful to conceptualize processes and think about treatments. Especially, cognitive rehabilitation methodologies urge a comprehensive theoretical framework that incorporates theories and models from different fields. The working memory model [11], the dual route model of reading [12], and the face recognition model [13] are examples of models that helped planning treatment for people with cognitive impairments. Nevertheless, until now, there is no single model or integrative cognitive rehabilitation framework that addresses the multiple aspects of cognitive functions involved in real life [14].

Although paper-and-pencil tasks are reliable tools to assess multiple domains of cognitive functioning (specific task scores can be used to evaluate the capacities of a patient in multiple cognitive domains) [15], there are few solutions to the inverse problem: a set of paper-and-pencil tasks that are specifically adapted to the results of different assessments of cognitive functioning of a patient [16,17]. Cognitive rehabilitation approaches have been relatively successful for focal cortical deficits (eg, neglect and aphasia) but less so for more generalized cognitive impairment (eg, slowed information processing and executive dysfunction) [5]. Additional research is needed to investigate the patient characteristics that influence treatment effectiveness [18]. Consequently, cognitive rehabilitation is still mostly planned and delivered based on the experience of the health professional and based on a subjective selection of paper-and-pencil cognitive tasks or conventional games, which are generally not adjusted to or validated for the specific cognitive needs of the patient [19]. Although we know that stroke-related cognitive problems are weighted more toward attention executive dysfunction than memory dysfunction and that there are marked deficits in abstraction, executive function, and processing speed [20], the cognitive impairment profile of each patient is highly variable and depends on the characteristics of his lesion.

### The Impact of Cognitive Rehabilitation on the Improvement of Cognitive Performance in Everyday Life

The American Congress of Rehabilitation Medicine conducted systematic reviews on a total of 370 studies about cognitive rehabilitation for people with traumatic brain injury (TBI) or stroke, published from 1971 through 2008 [21,22,18]. Cognitive rehabilitation was shown to be of greater benefit than conventional rehabilitation in 94.1% of the comparison studies. According to this evidence, there is a clear indication that cognitive rehabilitation is the best available form of treatment for people who exhibit cognitive impairments and functional limitations after TBI or stroke [18]. However, Paiva et al performed a meta-analysis on cognitive rehabilitation in stroke, and the results suggested a lack of sufficient evidence to support or refute the efficacy of cognitive interventions in stroke patients [23]. These divergent results should be interpreted with caution because in this meta-analysis, 504 of 507 studies were excluded because of low quality, and only 3 were considered by the authors. Additional research, using standardized assessment instruments and well-structured training programs, is needed to elucidate the mechanisms of change underlying the efficacy of cognitive rehabilitation.

The primary difficulty in determining the impact of cognitive interventions on the everyday functioning of healthy older adults is that most trials do not include functional outcome measures [24,25]. A review about the impact of cognitive training and mental stimulation on the cognitive and everyday functioning of healthy older adults from Kelly et al’s study (2014) found only 2 studies that examined the effects of cognitive training on everyday function [26]. One of them concluded that 6 months of memory training did not significantly improve everyday functioning for older adults at a 2-year follow-up [27], and the other study similarly reported no training effects on everyday functioning after 6 weeks of memory, reasoning, or processing speed training at a 2-year follow-up [28]. Interestingly, the later authors conducted a 5-year follow-up and concluded that successful performance in everyday tasks is critically dependent on executive cognitive function [29], which is supported by prior research that shows that the ability to perform independent living skills is dependent on intact executive function [30].

### Information and Communication Technologies

Over the past few years, several computer-based solutions have been proposed to increase the availability and quality of cognitive training, flooding the marketplace with commercial brain exercise programs that claim to improve cognition and have diagnostic abilities [31] such as the CogWeb [16,32,33] and the Guttmann Neuro Personal Trainer [34,35], for instance. There is also an increasing number of research projects focused in using a task modeling approach in poststroke rehabilitation, as the CogWatch, that developed intelligent common objects to help retraining Apraxia or action disorganization syndrome patients on how to carry out ADLs by providing persistent multimodal feedback to them [36]. Preliminary results involving 12 patients interacting with this system validated the ability of the system to assist stroke survivors in tea making. CogWatch was very beneficial to the patients who had difficulties performing the tasks alone, and when patients had access to the output retrieved by the system, their success rate was higher, and they made fewer errors than when they could not interact with the system.

Despite the proliferation of information and communication technologies (ICTs) in cognitive rehabilitation, only 5% to 15% of people with disabilities have access to technological devices that can assist in the rehabilitation process [37]. In addition, many health care providers are unfamiliar or uncomfortable with technology, and only about 27% of these professionals refer to use these computer-assisted technologies in their rehabilitation interventions [38]. Moreover, technological interventions are subject to continuous maintenance and technical support, eventually resulting in delayed interventions or the need to reschedule. Such complications speak to the challenges of implementing interventions dependent on technology within inpatient and outpatient rehabilitation settings. Any delays in these fast-paced settings, requiring the coordination of various professionals, can be disruptive [19].

To maximize the benefits of ICTs and to address the above-stated limitations, we developed a new Web-based tool, the Task Generator (TG). This Web tool capitalizes on the solid aspects of existing computerized training protocols for cognitive rehabilitation [17,32,39] and integrates existing theories and models [15]. The TG addresses multiple domains of cognitive functioning systematically and quantitatively, generating a profile of cognitive demands for each task and enabling the clinician to efficiently deliver a highly adapted training program to each patient’s deficits. The TG ultimately generates paper-and-pencil training tasks, making its application low cost and compatible with the current practice and existing limitations of clinical settings, and at the same time, it integrates most of the essential advantages of ICT-based interventions.

### Objectives

The objective of this research was to propose a systematic and objective design framework that can guide us on the methodology for the development of training tasks capable of addressing multiple domains of cognitive functioning, yet delivering a highly adaptive training program to each patient’s assessed deficits, and showcase its use in a Web-based app for cognitive rehabilitation.

## Methods

### Development Process

**Figure 1:**
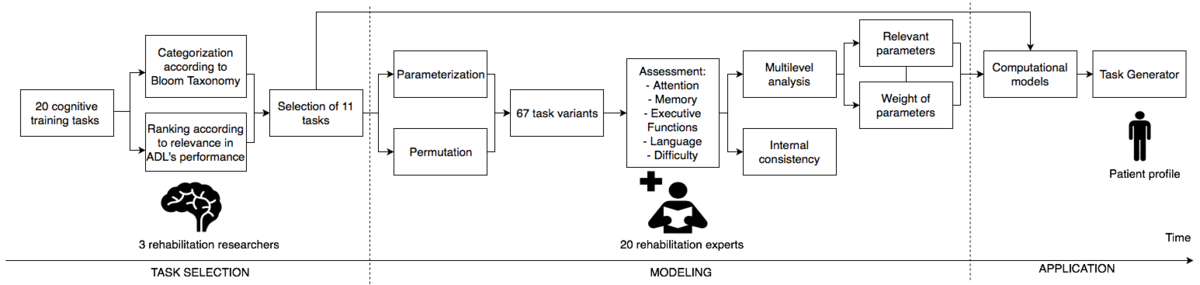
Methodology development process. ADLs: activities of daily living.

We have based our methodology on a participatory design strategy involving rehabilitation experts interworking with the research and development team through interviews, meetings, and questionnaires. In Figure 1, we describe the process we followed to identify and develop a set of highly personalized cognitive training tasks for a specific clinical group, in this case, stroke patients. It involved 3 main participatory steps: task selection, modeling, and application. However, the process followed is not unique to stroke rehabilitation and generalizes to any application area and target group where personalization of training is of importance.

#### Task Selection

As a first step toward the creation of a repertoire of cognitive training tasks, 3 rehabilitation experts (2 neuropsychologists with experience in cognitive assessment and interventions in stroke and dementia and an experienced rehabilitation technology researcher) documented the currently used methodologies in clinical rehabilitation settings (public hospitals, private clinics, and senior houses) and collected the most commonly used training tasks, some of them being available as published training material [40]. Of this search, 20 distinct paper-and-pencil task types were identified and analyzed.

As stated previously, no clear or comprehensive cognitive rehabilitation framework can provide us with general guidelines for cognitive training task selection. In the education field, however, there are multiple frameworks, the Bloom Taxonomy is one of the most relevant ones [41]. Hence, we have chosen and categorized the 20 tasks according to Bloom learning objectives as described below:

- *Knowledge (lower level):* memory of stories; cancellation; questions of general knowledge; find locations; image pairs
- *Comprehension:* differences between similar scenarios; categorization; synonyms and antonyms; association
- *Application*: mazes; problem resolution; tangram; numeric sequences; navigation
- *Analysis*: action sequencing; visual memory; puzzles; word search
- *Evaluation (higher level)*: differentiation between coherent and incoherent situations; comprehension of contexts

After the identification and organization of the 20 tasks according to their learning objectives, the 3 rehabilitation experts proceeded to a ranking of the 20 available cognitive tasks according to its relevance in the successful performance of ADLs. This *Task Selection* process, according to the learning objective’s representativeness and the relevance for ADLs performance, resulted in the selection of the following 11 tasks: word search, problem resolution, numeric sequences, action sequencing, association, cancellation, categorization, comprehension of contexts, image pairs, mazes, and memory of stories.

#### Modeling

It is necessary to identify the relevant tasks to train a specific cognitive deficit (such as attention and memory) to define a proper rehabilitation program, but that is not sufficient. It is imperative also to consider the learner characteristics to design adapted training capable of providing as best as possible a personalized rehabilitation. In our case, the learners are stroke patients with different deficits that need to be rehabilitated through intensive and continuous training. There is then, no one-fits-all training program. There should be a uniquely adapted rehabilitation program for patients according to their assessment of the multiple domains of cognitive functioning. Currently, this adaptation process is generated through tacit knowledge based on the clinicians’ subjective experience—which is essential and results from years of training—but there is no explicit formulation of such knowledge. This implicit knowledge is valid and necessary; however, to generalize, we should be able to transform it in a set of objective guidelines that support the personalization of training to the characteristics of each patient. To obtain such a set of guidelines and an objective way of operationalizing the adaptation in the different cognitive tasks, we followed a participatory design strategy with the main stakeholders.

**Table 1:** List of training tasks, their objectives, and parameters subject to personalization.

Training task	Objective	Parameters
Word search	A number of words can be found up, down, forward, or diagonally in a pool of randomized letters.	Words number; clue words; and clue pictures
Problem resolution	Two types of problems are presented, numeric calculations or calculations based on textual descriptions of daily activities.	Type; operations number; ones; and tens
Numeric sequences	A numeric sequence is given, and the subject has to come up with the missing numbers.	Step; ascending; and missing; position
Action sequencing	A list of randomized actions needed for the execution of several activities of daily living is presented.	Actions number and task goal
Association	A number of randomized pairs of items need to be paired correctly.	Pairs number
Cancellation	Find a target stimulus in a pool of distractors.	Distractors; letters; numbers; targets; and arrangement
Categorization	Grouping items into their underlying categories. The categories must be guessed from the items.	Categories number and items number
Comprehension of contexts	Some images are given with some descriptions. Correct descriptions need to be identified.	Descriptions number
Image pairs	A number of pairs of images to be memorized are presented. They must be recalled after 30 min.	Number of pairs
Mazes	Finding the way out of a labyrinth.	Size
Memory of stories	Recalling information about a read story or a picture by answering questions about it.	Type; size; and questions

##### Task Parameterization

This step had as primary objective to break down each of the 11 previously selected cognitive training tasks and identify their main parameters or variables to quantify their effects regarding demands in different domains of cognitive functioning. For that, we operationalized all tasks into their task parameters (independent variables; IVs) to study their demands in 4 cognitive domains (attention, memory, language, and executive functions) and for their overall difficulty (dependent variables; DVs). The breakdown of the tasks is as follows and is summarized in Table 1:

1. Word search: A predetermined number of words can be found up, down, forward, or diagonally in a pool of randomized letters. Words can overlap so that a letter can be part of 2 or more words. This task was operationalized according to the *number of words* to find and the *existence of clues* provided to identify words (pictures, words, or none).
2. Problem resolution: Here, 2 t*ypes of problems are presented*, numeric calculations or calculations based on a textual description of daily activities. Problems vary according to the *number of operations* involved and the use of numbers with *ones* or *tens*.
3. Numeric sequences: A numeric sequence is given as a finite sequence of numbers, and the subject must come up with the missing numbers. The task can be operationalized according to the number of *missing numbers* (*1, 2, or 3*) in the sequence, their *position* in the sequence, and the *step size* between numbers.
4. Action sequencing: In this task, a list of randomized steps needed for the execution of several ADLs is presented. The task can be defined by the *number of steps* to be ordered and whether the *goal* of the task is explicitly mentioned or must be guessed.
5. Association: The task comprehends a *number* of randomized pairs of items. These items need to be paired correctly according to a logical relationship between them.
6. Cancellation: The purpose of cancellation tasks is to find predetermined target stimulus in a pool of distractor stimulus. Thus, we operationalized this task according to the *type of stimulus* (letters, black or colored symbols, or numbers), the *pool size*, and their *arrangement* (randomly organized or in a grid structure).
7. Categorization: This task consists of organizing different items into their underlying categories. The names of the categories are not given, it must be guessed from the item’s or object’s relationships. The task can be defined according to the *number of categories* and the number of *items*.
8. Comprehension of contexts: In this task, some images are given with *some descriptions*, with some being *incorrect descriptions*.
9. Image pairs: In this task, *a number of pairs* of images are presented to be memorized. They are recalled after 30 min.
10. Mazes: The task consists of a labyrinth type of puzzle through which one must find the way out. The task can be operationalized according to the maze *size*.
11. Memory of stories: The task consists of recalling information about a read story or a pictorial scenario by answering questions about it. Stories can be textual or pictorial (*type*) and can have several descriptive elements (*size*) and a variable *number of questions*.

##### Task Permutation

After the operationalization of the previously mentioned 11 tasks and the identification of their underlying parameters, multiple variants of each task were created to explore all parameter space. Because it is not feasible to study the complete permutation of all combinations of task parameters for all tasks (a minimum of 134), task parameters were selected and combined according to what was feasible to implement and could be mathematically modeled. Table 1 describes the parameter combinations that were selected. Overall, we created 67 variants of the above 11 tasks.

##### Assessment

Subsequently, we further involved in this study a total of 20 external rehabilitation experts (3 physiatrists, 5 neuropsychologists, and 12 rehabilitation therapists) from the private and public sectors in the autonomous region of Madeira and mainland Portugal. None of them was involved in the previous steps of the design process. The age range of participants was from 26 to 56 years (mean=40.05, SD=10.26), and the experts’ experience range was from 2 to 32 years (mean=16.40, SD=10.54). Participants were 85% (17/20) female.

Each of the 20 study participants rated each of the 67 task variants in a 1 to 10 Likert scale according to their assessment of the tasks’ demands on attention, memory, language, executive functions domains, and difficulty. Participants were provided with the questionnaires to be completed within a week and the order in which participants rated the variants, and the amount of time required to complete the 67 of them was not controlled.

## Results

### Internal Consistency

The internal consistency of each questionnaire was assessed through the *Cronbach alpha*, which reported consistency in the experts’ responses for all tasks (Multimedia Appendix 1).

### Quantification of the Cognitive Profile of the Tasks

An analysis of the ratings of the 20 rehabilitation experts’ answers was performed to proceed to the identification of the relevant task parameters and the quantification of their impact regarding cognitive demands via a computational modeling approach. We have used this computational approach because traditional multiple regression techniques treat the units of analysis as independent observations, which is not the case in our study. The computational modeling was performed with the *R 3.1.1* software (Bell Labs), through the multilevel analysis package, which provides tools to estimate a wide variety of within-group agreement and reliability measures and provides data manipulation functions to facilitate multilevel analyses such as the one presented here [42]. A descriptive analysis per cognitive domain and overall difficulty (Table 2) was performed with the *Statistical Package for the Social Sciences 20* (IBM SPSS Statistics 20).

**Table 2:** Mean, minimum, and maximum ratings per task variant in each domain and overall difficulty.

Training task	Memory	Executive functions	Attention	Language	Difficulty
Word search, mean (range)	5.52 (5.05-6.20)	6.04 (5.60-6.55)	6.93 (6.50-7.60)	5.65 (5.25-6.00)	6.37 (5.70-7.00)
Problem resolution, mean (range)	6.10 (6.10-6.10)	7.23 (7.15-7.30)	6.97 (6.90-7.05)	5.20 (4.65-5.75)	6.19 (5.35-7.20)
Numeric sequences, mean (range)	5.30 (5.00-5.60)	6.65 (6.50-6.80)	6.87 (6.65-7.10)	4.68 (4.45-4.90)	3.06 (1.38-4.50)
Action sequencing	4.72 (3.35-5.65)	4.79 (3.90-5.65)	5.35 (3.80-6.40)	4.83 (3.50-5.75)	4.74 (3.15-6.20)
Association	3.37 (2.65-4.25)	3.92 (3.40-4.35)	3.95 (3.00-4.95)	3.28 (3.00-3.85)	3.78 (3.10-4.90)
Cancellation	3.59 (2.60-4.50)	3.98 (2.95-5.00)	5.09 (4.05-6.15)	2.94 (2.25-3.60)	4.08 (2.85-5.05)
Categorization	3.60 (2.20-5.00)	4.43 (2.85-5.95)	4.18 (2.60-5.65)	3.87 (2.80-4.70)	4.22 (2.35-6.05)
Comprehension of contexts	2.63 (2.60-2.65)	3.25 (2.65-3.85)	3.40 (3.20-3.60)	3.95 (3.45-4.45)	2.93 (2.55-3.30)
Image Pairs	6.97 (5.85-8.40)	5.55 (4.75-6.40)	6.75 (5.75-8.10)	4.62 (3.90-5.45)	6.35 (4.90-7.95)
Mazes	3.87 (2.90-4.90)	5.17 (3.70-6.45)	5.23 (4.10-6.50)	3.28 (2.65-3.70)	4.63 (3.20-6.10)
Memory of stories	6.36 (4.40-7.70)	4.89 (3.25-6.15)	6.67 (4.90-7.90)	5.41 (4.15-6.65)	5.95 (3.85-7.40)

**Figure 2:**
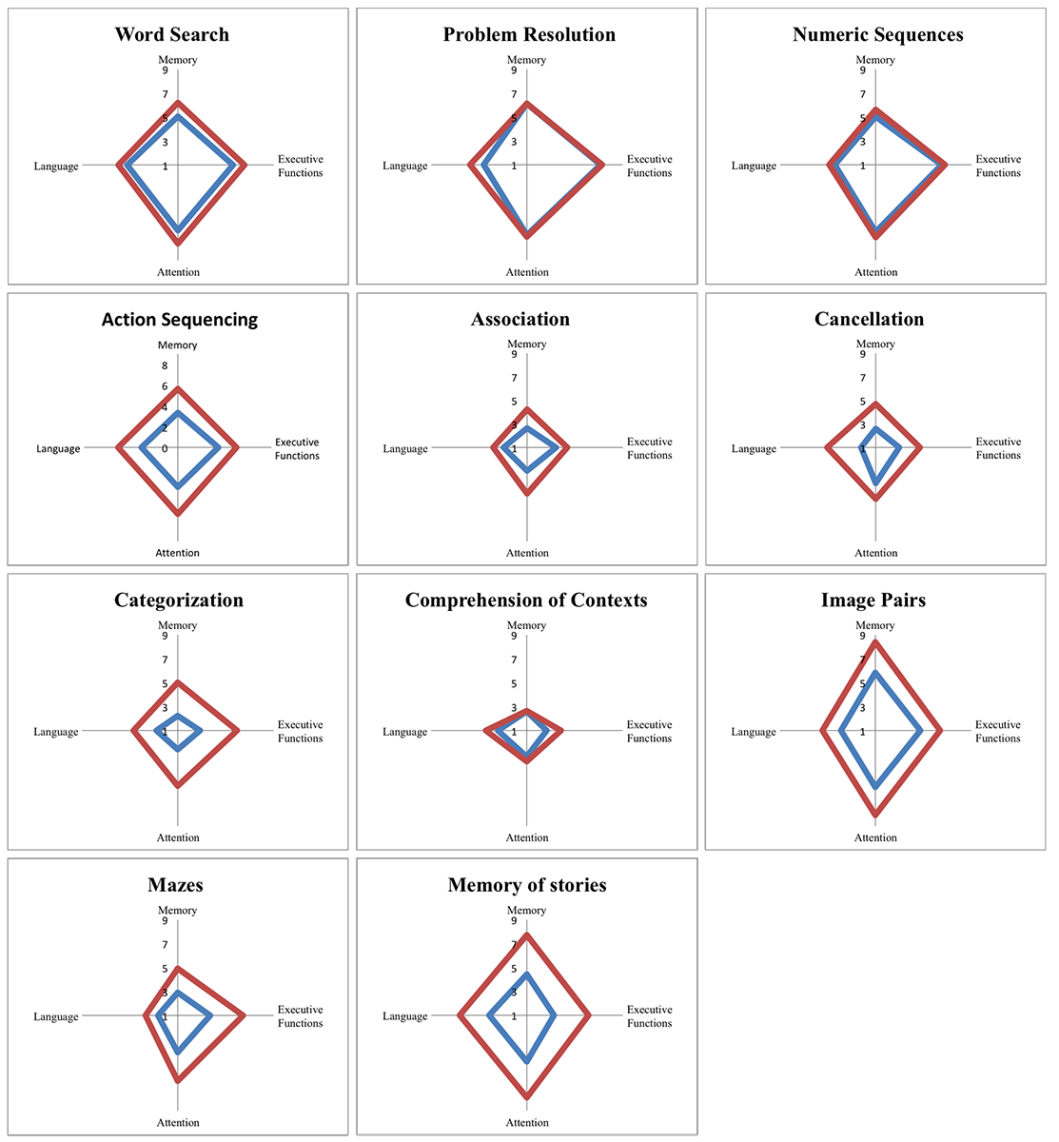
Task adaptation profiles represented as radar plots. Each plot has 4 axes—memory, executive functions, attention, and language—and the area between the blue (minimum) and the red line (maximum) represents the range interval in which the task varied depending on the selected task parameters in the study.

By assessing the minimum and maximum ratings per task variant in each domain, we can create a profile for every task, which is graphically represented in Figure 2, which determines each task’s training range. These profiles allow us to quickly judge the demands of each task and their adaptability in each cognitive domain. For instance, in the word search task, the demands range from 5.05 to 6.20 for memory, from 5.60 to 6.55 for the executive functions, from 6.50 to 7.60 for attention, and from 5.25 to 6 for language.

### Multilevel Analysis and Modeling

The above-reported ranges correspond to the ranges of the tested task variants, which are limited to the parameters described in Table 1. Through computational approaches, it is possible to further generalize these profiles by modeling the effect of untested parameters and combinations. Multilevel analysis was selected to accommodate the specificity of the data collected with partial observations (not all parameter combinations were assessed). The objective of the modeling approach was to quantitatively determine how the IVs (task parameters) impact each of the DVs (memory, executive functions, attention, language, and difficulty). To model this relationship, the parameters of each task (IVs) were used as predictors of the demands in each cognitive domain (DVs). A multilevel model of the following type was computed for each task:

DV=intercept+C1∗IV1+C2∗IV2+...+Ci∗IVi

where Ci indicates the contribution of each IV to the DV. These models considered a linear relationship with the order that the tasks were analyzed, allowed the slopes of these relationships to randomly vary, and incorporated an autoregressive structure with serial correlations in the error structures.

The basic procedure started by examining the nature of the outcome (task difficulty or cognitive load). First, we estimated the intraclass correlation coefficient and determined whether the outcome or DV (task difficulty or cognitive load) did not randomly vary among rehabilitation professionals. Thereafter, we considered only the significant IVs of the model. Second, we examined the form of the relationship between the order of the rated cognitive tasks and the outcome task difficulty or cognitive load. We wanted to know whether there was an order effect of the task’s rating. Third, we attempted to determine whether the relationship between the task order and the outcome or DVs is constant among individuals or whether it varies on an individual-by-individual basis. Fourth, we modeled the error structures such as autocorrelation [42].

The model quality was quantified, after each iteration, through the Akaike Information Criterion (AIC), Bayesian Information Criterion (BIC), and *P* values. AIC is an estimate of a constant plus the relative distance between the unknown true likelihood function of the data and the fitted likelihood function of the model so that a lower AIC means a model is considered to be closer to the truth. AIC does not provide a test of a model in the sense of testing a null hypothesis; therefore, it can tell nothing about the quality of the model in an absolute sense. BIC is an estimate of a function of the posterior probability of a model being true, under a specific Bayesian setup, so that a lower BIC means that a model is more likely to be the true model. Both criteria are based on various assumptions and asymptotic approximations. Hence, AIC and BIC provide a means for model selection. Each, despite its heuristic usefulness, has also been criticized as having questionable validity for real-world data. Our modeling process stopped at the step where the best model was generated according to AIC.

Through the computational analysis, we quantified how the manipulation of the IV impacted the DV. In some tasks and for some specific cognitive domains, it was not possible to model the relationship between IV and DV, which means that some parameter manipulations had no significant effects on the DV. In those cases, the mean rating is assumed in that domain. Task parameters that do not have a significant contribution to either of the cognitive domains or overall difficulty are omitted in the guidelines below. In the following, we present the detailed guidelines for the customization of training. Multimedia Appendices 2-10 and Tables 3-6 contain the mathematical models together with the AIC and BIC values, which helped us to determine if we should perform the third *(Order)* and fourth *(AutoCorr)* steps of the modeling process.

**Table 3:** Problem resolution task models for language and difficulty.

Problem resolution task		Language			Difficulty		
		Coefficient value	SE	*t* value	Coefficient value	SE	*t* value
Intercept		4.65	0.562	8.281	4.870	0.568	8.573
Type		1.10	0.242	4.548	—^a^	—	—
Operations number		—	—	—	0.542	0.080	6.737
Tens		—	—	—	0.365	0.186	1.964

^a^Not applicable.

**Table 4:** Problem resolution task models quality for language and difficulty.

Model Quality	Language	Difficulty
Akaike Information Criterion	645.2693	794.0537
Bayesian Information Criterion	668.2871	813.7529
Order	Yes	Yes
Autocorrelation	Yes	Yes

**Table 5:** Comprehension of contexts task models for executive functions, language and difficulty.

Comprehension of contexts task		Executive functions			Language			Difficulty		
		Coefficient value	SE	*t* value	Coefficient value	SE	*t* value	Coefficient value	SE	*t* value
Intercept		0.25	1.235	0.202	1.45	1.268	1.144	1.05	0.694	1.513
Descriptions number		1.20	0.457	2.629	1.00	0.453	2.207	0.75	0.228	3.290

**Table 6:** Comprehension of contexts task models quality for executive functions, language and difficulty.

Model quality	Executive functions	Language	Difficulty
Akaike Information Criterion	177.3641	184.2205	144.2994
Bayesian Information Criterion	183.9144	190.7708	150.8498
Order	No	No	No
Autocorrelation	No	No	No

#### Word Search (Impact Memory, Attention, and Executive Functions)

Through raising the number of words, it is possible to increase overall difficulty, memory, attention, and executive functions’ demands. In addition, if clues are given in images, it is more difficult and demanding for memory, attention, and executive functions (Multimedia Appendix 2).

#### Problem Resolution (Impact Language)

The task allows the training of language by presenting the problems through real daily living situations. A higher number of operations and number of digits increase the general difficulty of this task (Tables 3 and 4).

#### Numeric Sequences (Impact Memory, Attention, Executive Functions, and Language)

The higher the demands for training memory, attention, executive functions, and language, the more the missing numbers, and yet higher if they are omitted at the beginning of the sequence. Concerning overall difficulty, the task is more laborious if the sequence is in descending order and the higher the step size between the sequence numbers is (Multimedia Appendix 3).

#### Action Sequencing (Impact Memory, Attention, Executive Functions, and Language)

A higher number of steps are needed to increase the cognitive demands. Also, it is possible to make the training more demanding for attention and language if the task goal is not explicitly mentioned (Multimedia Appendix 4).

#### Association (Impact Memory, Attention, Executive Functions, and Language)

Augmenting the number of pairs will increase the difficulty as well as the training of memory, attention, executive functions, and language (Multimedia Appendix 5).

#### Cancellation (Impact Memory, Attention, Executive Functions, and Language)

Memory and attention demands can be increased by using symbols and letters instead of numbers and by having more distractors and targets. For training in the language domain, we should use symbols and increase the number of distractors. By increasing both targets and distractors and using symbols, the task gets more difficult and more demanding in executive functions (Multimedia Appendix 6).

#### Categorization (Impact Memory, Attention, Executive Functions, and Language)

Augmenting the number of categories will increase the difficulty of the task as well as the training of memory, executive functions, and language. Concerning attention, besides augmenting the number of categories, we need to have more items per category (Multimedia Appendix 7).

#### Comprehension of Contexts (Impact Executive Functions and Language)

The higher the number of descriptions per context, the higher the demands for executive functions, language, and difficulty (Tables 5 and 6).

#### Image Pairs (Impact Memory, Attention, Executive Functions, and Language)

Increasing the number of images to pair will increase the difficulty of the task and the training of memory, attention, executive functions, and language (Multimedia Appendix 8).

#### Mazes (Impact Memory, Attention, Executive Functions, and Language)

They can be used to train memory, attention, executive functions, and language. By augmenting the size of the mazes, the cognitive demands and general difficulty are increased (Multimedia Appendix 9).

#### Memory of Stories (Impact Memory, Attention, Executive Functions, and Language)

To increase demands for memory, attention, and general difficulty, we need to increase the length of the story and the number of questions about it. To train executive functions and language, increasing the story length is enough (Multimedia Appendix 10).

The above modeling effort of the selected cognitive training tasks—selected for their high impact in the realization of ADLs—enables us to create a cognitive rehabilitation program that is precisely adjusted to each individual cognitive domain depending on the specific profile of each patient in terms of memory, attention, executive functions, language demands, and overall difficulty. Our computational approach, thus, captures the implicit rehabilitation experts’ experience and knowledge quantitatively; thus, providing us with explicit models to create an adaptation engine capable of personalizing cognitive training.

### App: the Task Generator

Still today, paper-and-pencil tasks are the most widely used means of cognitive rehabilitation [43] because of their acceptance, clinical validity, and reduced cost [44]. However, one of their limitations is that they lack flexibility and personalization. Consequently, it would be advantageous to have a tool that could generate standard, accepted, and validated paper-and-pencil tasks, yet customized according to any patient profile. This approach would mitigate some of the most critical limitations of paper-and-pencil tasks. For this reason, we have created a free and world-accessible Web-based tool, the TG, for the generation of personalized cognitive training tasks (see Multimedia Appendix 11). The TG is a Web-based app and does not require to be installed on the computer; the only software required is a PDF reader to open the downloaded files. Through this tool, clinicians can define appropriate parameters of training for memory, attention, executive functions, language, and difficulty, and it automatically generates the requested personalized cognitive training tasks based on the task adaptation profiles represented as radar plots in Figure 2 (the area between the minimum and the maximum line represents the range interval in which each task can vary).

**Figure 3:**
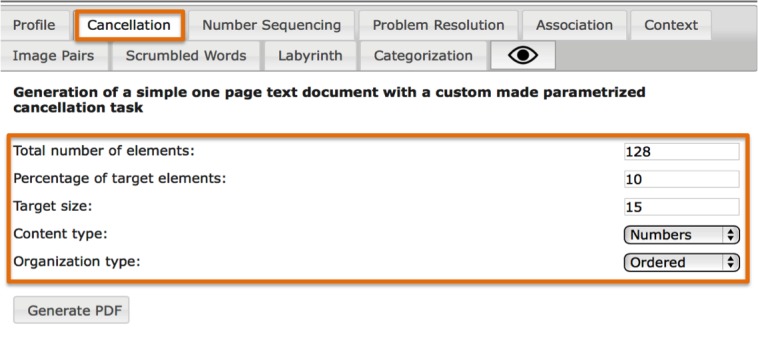
Individual tasks can also be generated by specifying the value of their parameters (cancellation task example).

**Figure 4:**
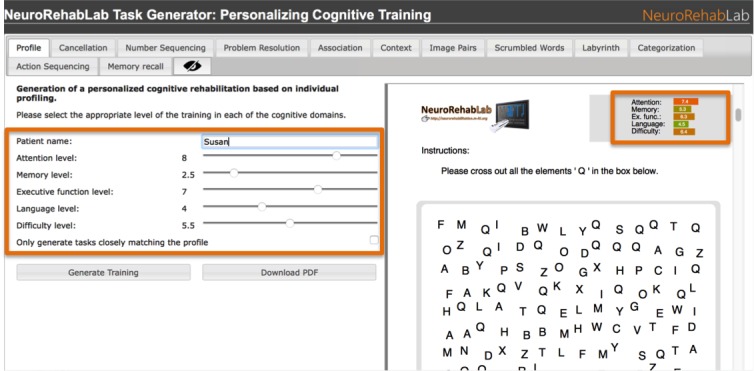
A cognitive training program can be generated by specifying the intended training intensity in each cognitive domain. Each training task contains a visual task profile, indicating its demands in attention, memory, executive functions, language, and difficulty.

**Figure 5:**
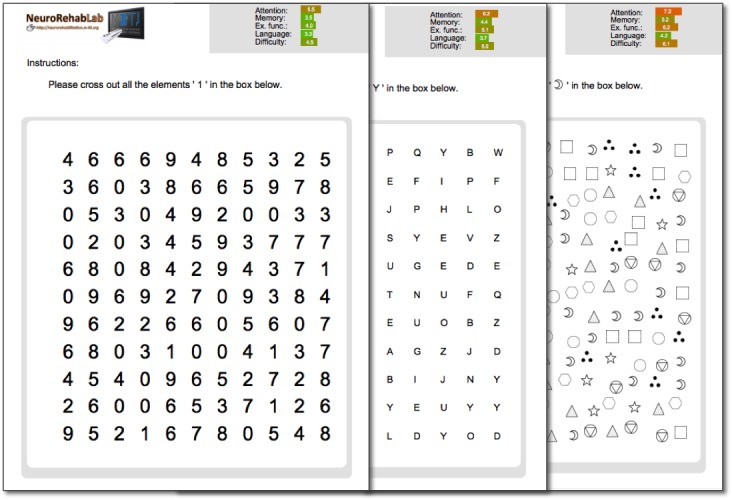
Example of different parameterizations of the cancellation task. The graphical profile changes according to the parameters defined by the clinician.

Tasks can be created either individually by directly specifying the values of their parameters (Figure 3) or as a full cognitive training program containing the whole set of 11 personalized training tasks. Tasks are created procedurally; 2 training tasks are never the same, allowing for the repeated use of this tool. Besides, the generated tasks have a task profile (Figure 4)—a graphical representation of their demands in each cognitive domain and difficulty—enabling clinicians to efficiently and continuously adapt the training to the patient’s needs (Figure 5).

### Training Adaptation Over Time

When the patient finishes a set of tasks, the clinician may use one of these 2 procedures:

1. *From training session to training session*: By scoring the TG task’s performance using a 0% to 100% scale and computing the mean performance of the whole task’s set. If the mean performance is higher than a specific threshold (for instance, assuming an optimal performance from 70% to 100% [45]), the clinician should increase by 0.5 only the difficulty parameter while keeping the ones related to memory, attention, executive functions, and language constant. Alternatively, if performance is from 0% to 50%, the difficulty parameter should be reduced by 0.5.
2. *After a progress evaluation point*: By performing a new assessment of the patient profile. A new set of training tasks is generated with the new assessment following the same procedure stated in the *Cognitive Training Program Generation* section.

### Full Cognitive Training Program Generation

Once a patient is assessed, and the patient’s deficits and cognitive profile are known, the clinician’s challenge is that of adapting the available training tasks to this patient. TG solves that problem by allowing clinicians to quickly generate a complete cognitive training program, containing the whole set of 11 tasks by simply specifying the cognitive profile for a patient in 4 cognitive domains (memory, attention, executive functions, and language), and the overall task difficulty in a 1 to 10 scale. This can be easily done through the characterization of the patient with validated instruments such as the Montreal Cognitive Assessment (MoCA) [46]. The TG *Attention* parameter can be defined from MoCA’s attention component score (0-6); the delayed recall and orientation scores (0-11) can be used to parameterize *memory; executive functions* can be parameterized through the sum of the visuospatial, executive, and abstraction MoCA subscores (0-7); MoCA’s naming and the language scores (0-6) can be used to parameterize *language;* and the total score (0-30) can be used to parameterize the overall *difficulty.* After the characterization of a patient, through the normalization of these assessment results on a 1 to 0 scale, a full training program is generated by pressing the *Generate Training* button and then can be downloaded as a PDF file by pressing the *Download PDF* button. In addition, there is an optional check box in the patient profile page that when selected only generates tasks closely matching the chosen profile. Tasks that would differ substantially from the selected profile can then be filtered out as they can represent nonoptimal task parameter choices. Nonetheless, the user can disable this feature by unchecking the selection box and the TG will generate the complete set of 11 tasks, with the best possible personalization allowed by their parameters.

## Discussion

### Principal Findings

We developed a design framework where we borrowed concepts from educational psychology and a participatory design strategy with stakeholders to support the development process. Through this process, we were able to identify a representative group of well-established standard paper-and-pencil tasks currently used for cognitive rehabilitation, and we operationalized them with respect to their parameters. To that end, the expert knowledge of 20 rehabilitation experts was used to model each task for its difficulty and impact on cognitive functions. The task models obtained provide us with valuable guidelines toward the development of personalized cognitive rehabilitation tools. Furthermore, we demonstrated the proposed methodology with an example case: a Web-based tool for the generation of customized paper-and-pencil cognitive training tasks, the TG. We believe that the TG contributes toward the definition of objective procedures for the application of adaptive cognitive rehabilitation through the use of ICTs. The use of TG has virtually zero cost associated, and it is available in English, Portuguese, and Italian.

### Comparison With Prior Work

Recent technological advances have allowed improved apps for cognitive rehabilitation, and it has been shown that they can be effective rehabilitation tools for health professionals [33]. However, the lack of a precise design methodology that can guide the development of ICT’s applications, applied to rehabilitation, still remains one of the main limitations in this field. Data mining techniques have been applied to predict the outcomes of cognitive rehabilitation in patients with acquired brain injury; however, rehabilitation experts’ input should also be included [47]. As an answer to this need, the primary goal of this study was to propose a general framework to guide in the design of future cognitive rehabilitation tools, with objective and expert-based guidelines.

The app here presented guidelines in a Web-based tool as the TG also addresses the accessibility limitations because it can be widely deployed at health care centers and home. This new approach does not interfere with current clinical practices because it produces printable paper-and-pencil tasks. By enabling the adaptation of task parameters and difficulty levels according to patient performance, this tool provides a comprehensive and highly personalized cognitive training.

### Limitations

Despite the valuable guidelines obtained, via computational modeling, from our participatory design strategy, some limitations of our study must be considered. First, there is a considerable variety of paper-and-pencil tasks being used in cognitive rehabilitation and stimulation practice, and we have selected a small subset of 11 tasks to be possible to parameterize and present them in a questionnaire; however, we are aware it is a small number. Second, concerning the sample of rehabilitation experts, 20 participants can be considered a small number although we managed to include different professionals: physicians, psychologists, and therapists. Third and last, our participatory design strategy was limited in the sense that we did not include subjective and qualitative feedback from the rehabilitation experts, except for one of the physiatrists who was involved in the task selection phase.

### Developments of This Study

Although paper-and-pencil tasks are widely used in cognitive rehabilitation, these tools mostly focus on isolated components of cognitive functioning, which have been reported to disagree with everyday life tasks [44,48]. It has been shown that virtual reality (VR), as a tool, has a significant potential for enhancing the reliability and specificity of cognitive assessment and rehabilitation [19,49]. Due to all the VR advantages, the logical next step is the integration of the computational models obtained through the participatory design study in a cognitive VR rehabilitation environment presented here. In this context, we integrated the findings from our models and transformed the original paper-and-pencil tasks in virtual ADL's tasks within a simulation of a city with streets, sidewalks, realistic buildings, several parks, and moving vehicles—the Reh@City [50]. The activities in the Reh@City are organized in parameterized difficulty levels and target the cognitive domains addressed in the guidelines presented here: memory, attention, executive functions, and language. As an illustrative example, in terms of attention, Reh@City incorporates relevant ADL's, implementation of which helps bridge paper-and-pencil cancellation tasks. More specifically, targets and distractors are embedded in a pharmacy, a supermarket, or a post-office shelf. This kind of implementation allows the operationalization of the training difficulty by changing the number and nature of targets and distractors, their sizes, and their spatial arrangement.

Currently, we are running a 1-month longitudinal randomized controlled trial comparing both TG and Reh@City v2.0 interventions. This study entails a comprehensive neuropsychological assessment not only pre- and post intervention but also at follow-up, with the aim of comparing the impact of a personalized paper-and-pencil program (TG), a personalized and integrative VR-based program (Reh@City v2.0), and conventional therapy. The main objective of this study was to assess the neuropsychological and functional impact of a paper-and-pencil task and a VR intervention, having the same tasks and parameterization guidelines for comparison. In addition, in this study, we are also addressing the usability of the tool through interviews and questionnaires so that we can improve both tools regarding the patients’ perspective.

### Future Work

Many health care providers are unfamiliar with ICTs and, as a consequence, a very small percentage of people with disabilities have access to technological devices that can assist them in the rehabilitation process. To mitigate this issue, it would be valuable to improve the usability of both the TG and the Reh@City by interviewing the health care providers after using them as complementary tools for their work.

Moreover, as future work, we are also planning to upgrade the TG app by creating a tablet version that allows remote monitoring by the health care providers and automatic personalization through artificial intelligence and machine learning algorithms.

## Appendix

Multimedia Appendix 1 Questionnaire’s internal consistency.

Multimedia Appendix 2 Word search task models for memory, attention, executive functions and difficulty.

Multimedia Appendix 3 Numeric sequences task models for memory, attention, executive functions, language, and difficulty.

Multimedia Appendix 4 Action sequencing task models for memory, attention, executive functions, language, and difficulty.

Multimedia Appendix 5 Association task models for memory, attention, executive functions, language, and difficulty.

Multimedia Appendix 6 Cancellation task models for memory, attention, executive functions, language, and difficulty.

Multimedia Appendix 7 Categorization task models for memory, attention, executive functions, language, and difficulty.

Multimedia Appendix 8 Image pairs task models for memory, attention, executive functions, language, and difficulty.

Multimedia Appendix 9 Mazes task models for memory, attention, executive functions, language, and difficulty.

Multimedia Appendix 10 Memory of stories and images task models for memory, attention, executive functions, language and difficulty.

Multimedia Appendix 11 Task Generator video.

## Abbreviations

ADL: activity of daily living
AIC: Akaike Information Criterion
BIC: Bayesian Information Criterion
DV: dependent variable
ICT: information and communication technology
IV: independent variable
MoCA: Montreal Cognitive Assessment
TBI: traumatic brain injury
TG: Task Generator
VR: virtual reality
