# Supplementary material for: Capturing Expert Knowledge for the Personalization of Cognitive Rehabilitation: Study Combining Computational Modeling and a Participatory Design Strategy
Source: JMIR Rehabil Assist Technol. 2018 Dec 6;5(2):e10714. doi: 10.2196/10714 (PMC6318149; doi:10.2196/10714)
Supplement: Multimedia Appendix 1 [file rehab_v5i2e10714_app1.pdf]

Tasks	Word search	Problem resolution	Numeric sequences	Action sequencing	Association	Cancellation	Categorization	Contexts	Image pairs	Mazes	Memory of stories
Alpha	0.981	0.987	0.997	0.973	0.975	0.990	0.953	0.945	0.963	0.975	0.918
