# Supplementary material for: Capturing Expert Knowledge for the Personalization of Cognitive Rehabilitation: Study Combining Computational Modeling and a Participatory Design Strategy
Source: JMIR Rehabil Assist Technol. 2018 Dec 6;5(2):e10714. doi: 10.2196/10714 (PMC6318149; doi:10.2196/10714)
Supplement: Multimedia Appendix 2 [file rehab_v5i2e10714_app2.pdf]

Word search task	Memory			Attention			Executive functions			Difficulty		
	Coefficient value	Standard error	*t* value	Coefficient value	Standard error	*t* value	Coefficient value	Standard error	*t* value	Coefficient value	Standard error	*t* value
Intercept	4.000	0.752	5.320	6.150	0.617	9.968	5.138	0.715	7.187	5.466	0.688	7.947
Clue words	-1.000	0.346	-2.893	-.700	0.327	-2.142	-0.913	0.229	-3.993	-1.154	0.287	-4.013
Words number	0.269	0.071	3.809	0.144	0.067	2.155	0.171	0.054	3.169	0.176	0.069	2.561

Model quality	Memory	Attention	Executive functions	Difficulty
Akaike Information Criterion	403.9686	384.4461	345.6128	373.7182
Bayesian Information Criterion	416.8422	397.3196	363.6358	391.7411
Order	No	No	Yes	Yes
Autocorrelation	No	No	No	No
