# Supplementary material for: Capturing Expert Knowledge for the Personalization of Cognitive Rehabilitation: Study Combining Computational Modeling and a Participatory Design Strategy
Source: JMIR Rehabil Assist Technol. 2018 Dec 6;5(2):e10714. doi: 10.2196/10714 (PMC6318149; doi:10.2196/10714)
Supplement: Multimedia Appendix 3 [file rehab_v5i2e10714_app3.pdf]

Numeric sequences task	Memory			Attention			Executive functions			Language			Difficulty		
	Coefficient value	Standard error	*t* value	Coefficient value	Standard error	*t* value	Coefficient value	Standard error	*t* value	Coefficient value	Standard error	*t* value	Coefficient value	Standard error	*t* value
Intercept	5.364	0.700	7.667	6.923	0.528	13.103	6.682	0.570	11.715	4.722	0.630	7.497	1.290	0.515	2.506
Step	___	___	___	___	___	___	___	___	___	___	___	___	1.232	0.126	9.750
Ascending	___	___	___	___	___	___	___	___	___	___	___	___	-0.841	0.154	-5.457
Missing	-0.027	0.009	-3.142	-0.020	0.006	-3.342	-0.014	0.005	-2.818	-0.020	0.007	-3.100	___	___	___
Position	-0.003	0.001	-2.835	-0.003	0.001	-3.017	-0.002	0.001	-2.546	-0.003	0.001	-2.799	___	___	___

Model quality	Memory	Attention	Executive functions	Language	Difficulty
Akaike Information Criterion	177.0194	-97.91632	-272.89619	-34.42772	480.4425
Bayesian Information Criterion	208.8909	-66.04483	-241.02470	-2.55623	504.8925
Order	Yes	Yes	Yes	Yes	Yes
Autocorrelation	Yes	Yes	Yes	Yes	Yes
