# Supplementary material for: Capturing Expert Knowledge for the Personalization of Cognitive Rehabilitation: Study Combining Computational Modeling and a Participatory Design Strategy
Source: JMIR Rehabil Assist Technol. 2018 Dec 6;5(2):e10714. doi: 10.2196/10714 (PMC6318149; doi:10.2196/10714)
Supplement: Multimedia Appendix 4 [file rehab_v5i2e10714_app4.pdf]

Action sequencing task	Memory			Attention			Executive functions			Language			Difficulty		
	Coefficient value	Standard error	*t* value	Coefficient value	Standard error	*t* value	Coefficient value	Standard error	*t* value	Coefficient value	Standard error	*t* value	Coefficient value	Standard error	*t* value
Intercept	1.507	0.669	2.254	2.90	0.691	4.197	2.838	0.911	3.113	3.325	0.812	4.096	1.950	0.664	2.937
Actions number	0.635	0.153	4.159	0.75	0.125	5.988	0.487	0.202	2.409	0.525	0.146	3.601	0.862	0.124	6.966
Task goal	___	___	___	-1.10	.251	-4.391	___	___	___	-1.200	0.292	-4.115	-1.325	0.248	-5.351

Model quality	Memory	Attention	Executive functions	Language	Difficulty
Akaike Information Criterion	334.9841	307.1238	358.3382	331.2999	302.795
Bayesian Information Criterion	349.1244	318.8428	367.7650	343.0189	314.5140
Order	Yes	No	No	No	No
Autocorrelation	No	No	No	No	No
