# Supplementary material for: Capturing Expert Knowledge for the Personalization of Cognitive Rehabilitation: Study Combining Computational Modeling and a Participatory Design Strategy
Source: JMIR Rehabil Assist Technol. 2018 Dec 6;5(2):e10714. doi: 10.2196/10714 (PMC6318149; doi:10.2196/10714)
Supplement: Multimedia Appendix 5 [file rehab_v5i2e10714_app5.pdf]

Association task	Memory			Attention			Executive functions			Language			Difficulty		
	Coefficient value	Standard error	*t* value	Coefficient value	Standard error	*t* value	Coefficient value	Standard error	*t* value	Coefficient value	Standard error	*t* value	Coefficient value	Standard error	*t* value
Intercept	1.367	0.782	1.747	1.513	0.721	2.099	2.729	0.721	3.788	2.221	0.403	5.511	1.533	0.701	2.186
Pairs number	0.400	0.117	3.426	0.487	0.100	4.892	0.237	0.088	2.701	0.106	0.038	2.816	0.450	0.104	4.323

Model quality	Memory	Attention	Executive functions	Language	Difficulty
Akaike Information Criterion	265.1870	252.8099	225.6666	213.2304	252.3468
Bayesian Information Criterion	273.4288	261.0517	238.0292	225.5931	260.5886
Order	No	No	Yes	Yes	No
Autocorrelation	No	No	No	No	No
