# Supplementary material for: Capturing Expert Knowledge for the Personalization of Cognitive Rehabilitation: Study Combining Computational Modeling and a Participatory Design Strategy
Source: JMIR Rehabil Assist Technol. 2018 Dec 6;5(2):e10714. doi: 10.2196/10714 (PMC6318149; doi:10.2196/10714)
Supplement: Multimedia Appendix 6 [file rehab_v5i2e10714_app6.pdf]

Cancellation task	Memory			Attention			Executive functions			Language			Difficulty		
	Coefficient value	Standard error	*t* value	Coefficient value	Standard error	*t* value	Coefficient value	Standard error	*t* value	Coefficient value	Standard error	*t* value	Coefficient value	Standard error	*t* value
Intercept	3.125	0.505	6.193	4.314	0.532	8.103	3.459	0.504	6.862	2.839	0.497	5.717	3.610	0.514	7.018
Distractors	0.009	0.003	2.959	0.014	0.003	4.327	0.014	0.003	4.087	0.009	0.003	3.383	0.015	0.003	4.601
Letters	___	___	___	___	___	___	-0.814	0.211	-3.853	-0.480	0.178	-2.701	-0.494	0.210	___
Numbers	-0.813	0.214	3.126	-0.697	0.235	-2.969	-0.845	0.236	-3.587	-0.790	0.204	-3.869	-1.054	0.240	-4.385
Targets	0.017	0.005	-3.802	0.021	0.006	3.526	0.012	0.005	2.150	___	___	___	0.012	0.006	2.115
Arrangement	___	___	___	___	___	___	0.724	0.266	2.716	___	___	___	___	___	___

Model quality	Memory	Attention	Executive functions	Language	Difficulty
Akaike Information Criterion	738.4285	773.6644	743.1042	691.5384	749.9689
Bayesian Information Criterion	758.0972	793.3330	772.5149	714.4852	776.1529
Order	No	No	Yes	Yes	Yes
Autocorrelation	No	No	Yes	Yes	Yes
