# Supplementary material for: Capturing Expert Knowledge for the Personalization of Cognitive Rehabilitation: Study Combining Computational Modeling and a Participatory Design Strategy
Source: JMIR Rehabil Assist Technol. 2018 Dec 6;5(2):e10714. doi: 10.2196/10714 (PMC6318149; doi:10.2196/10714)
Supplement: Multimedia Appendix 7 [file rehab_v5i2e10714_app7.pdf]

Categorization	Memory			Attention			Executive functions			Language			Difficulty		
task	Coefficient value	Standard error	*t* value	Coefficient value	Standard error	*t* value	Coefficient value	Standard error	*t* value	Coefficient value	Standard error	*t* value	Coefficient value	Standard error	*t* value
Intercept	0.6	0.684	0.877	-3.26	1.981	-1.645	1.136	0.695	1.635	1.914	0.724	2.644	0.234	0.462	0.506
Categories number	0.9	0.156	5.754	3.75	1.262	2.971	0.989	0.148	6.702	0.586	0.151	3.871	1.165	0.145	8.048
Items number	___	___	___	-0.41	0.185	-2.213	___	___	___	___	___	___	___	___	___

Model quality	Memory	Attention	Executive functions	Language	Difficulty
Akaike Information Criterion	260.2888	233.4358	259.6256	263.7335	244.8731
Bayesian Information Criterion	268.5306	243.6511	267.8674	271.9753	257.2357
Order	No	No	No	No	Yes
Autocorrelation	No	No	No	No	No
