# Supplementary material for: Capturing Expert Knowledge for the Personalization of Cognitive Rehabilitation: Study Combining Computational Modeling and a Participatory Design Strategy
Source: JMIR Rehabil Assist Technol. 2018 Dec 6;5(2):e10714. doi: 10.2196/10714 (PMC6318149; doi:10.2196/10714)
Supplement: Multimedia Appendix 8 [file rehab_v5i2e10714_app8.pdf]

Image Pairs task	Memory			Attention			Executive Functions			Language			Difficulty		
	Coefficient value	Standard error	*t* value	Coefficient value	Standard error	*t* value	Coefficient value	Standard error	*t* value	Coefficient value	Standard error	*t* value	Coefficient Value	Standard error	*t* value
Intercept	3.779	0.600	6.293	3.813	0.660	5.774	3.488	0.659	5.290	2.723	0.809	3.367	2.538	0.615	4.124
Number of pairs	0.637	0.081	7.867	0.587	0.096	6.123	0.412	0.081	5.062	0.388	0.114	3.409	0.762	0.092	8.276

Model quality	Memory	Attention	Executive Functions	Language	Difficulty
Akaike Information Criterion	230.5643	244.6216	236.9498	250.4309	237.4246
Bayesian Information Criterion	238.8061	252.8633	245.1916	260.7332	245.6663
Order	No	No	No	Yes	No
Autocorrelation	No	No	No	Yes	No
