# Supplementary material for: Capturing Expert Knowledge for the Personalization of Cognitive Rehabilitation: Study Combining Computational Modeling and a Participatory Design Strategy
Source: JMIR Rehabil Assist Technol. 2018 Dec 6;5(2):e10714. doi: 10.2196/10714 (PMC6318149; doi:10.2196/10714)
Supplement: Multimedia Appendix 9 [file rehab_v5i2e10714_app9.pdf]

Mazes task	Memory			Attention			Executive functions			Language			Difficulty		
	Coefficent value	Standard error	*t* value	Coefficent value	Standard error	*t* value	Coefficent value	Standard error	*t* value	Coefficent value	Standard error	*t* value	Coefficent value	Standard error	*t* value
Intercept	1.867	0.475	3.932	2.876	0.612	4.701	2.390	0.678	3.527	2.233	0.416	5.364	1.733	0.628	2.759
Size	1.000	0.221	4.532	1.200	0.186	6.460	1.375	0.217	6.328	0.525	0.198	2.655	1.450	0.188	7.706

Model quality	Memory	Attention	Executive functions	Language	Difficulty
Akaike Information Criterion	245.2409	230.2526	249.9474	232.7764	245.6437
Bayesian Information Criterion	257.6036	238.4944	258.1892	245.1390	253.8855
Order	Yes	No	No	Yes	No
Autocorrelation	No	No	No	No	No
