# Supplementary material for: Capturing Expert Knowledge for the Personalization of Cognitive Rehabilitation: Study Combining Computational Modeling and a Participatory Design Strategy
Source: JMIR Rehabil Assist Technol. 2018 Dec 6;5(2):e10714. doi: 10.2196/10714 (PMC6318149; doi:10.2196/10714)
Supplement: Multimedia Appendix 10 [file rehab_v5i2e10714_app10.pdf]

Memory of stories task	Memory			Attention			Executive functions			Language			Difficulty		
	Coefficient value	Standard error	*t* value	Coefficient value	Standard error	*t* value	Coefficient value	Standard error	*t* value	Coefficient value	Standard error	*t* value	Coefficient value	Standard error	*t* value
Intercept	3.60	0.561	6.413	4.2	0.644	6.517	1.90	0.705	2.693	3.083	0.726	4.248	2.800	0.529	5.295
Size	4.20	1.011	4.155	3.9	0.822	4.746	1.45	0.278	5.216	1.250	0.265	4.718	3.950	0.982	4.024
Questions	-0.85	0.331	-2.569	-0.8	0.263	-3.039	___	___	___	___	___	___	-0.725	0.321	-2.257

Model quality	Memory	Attention	Executive functions	Language	Difficulty
Akaike Information Criterion	234.0854	237.5185	270.4890	271.7985	227.5656
Bayesian Information Criterion	244.3007	249.7768	278.7308	280.0402	237.7809
Order	No	No	No	No	No
Autocorrelation	No	No	No	No	No
